# Importance analysis of psychosociological variables in frailty syndrome in heart failure patients using machine learning approach

**DOI:** 10.1038/s41598-023-35037-3

**Published:** 2023-05-13

**Authors:** Aleksandra Helena Pasieczna, Remigiusz Szczepanowski, Janusz Sobecki, Radosław Katarzyniak, Izabella Uchmanowicz, Robbert J. J. Gobbens, Aleksander Kahsin, Anant Dixit

**Affiliations:** 1 University of Lower Silesia DSW, Wrocław, Poland; 2 Department of Computer Science and Systems Engineering, Faculty of Information and Communication Technology, Wroclaw University of Science and Technology, Wyb. Wyspiańskiego 27, 50-370 Wroclaw, Poland; 3 Department of Nursing and Obstetrics, Faculty of Health Sciences, Wroclaw Medical University, Wroclaw, Poland; 4 Faculty of Health, Sports and Social Work, Inholland University of Applied Sciences, Amsterdam, the Netherlands; 5 Department Family Medicine and Population Health, Faculty of Medicine and Health Sciences, University of Antwerp, Antwerp, Belgium; 6 Tranzo, Tilburg School of Social and Behavioral Sciences, Tilburg University, Tilburg, the Netherlands; 7 Zonnehuisgroep Amstelland, Amstelveen, the Netherlands; 8 Faculty of Medicine, Medical University of Gdansk, Gdansk, Poland

**Keywords:** Cardiology, Health care, Medical research, Engineering, Mathematics and computing

## Abstract

The prevention and diagnosis of frailty syndrome (FS) in cardiac patients requires innovative systems to support medical personnel, patient adherence, and self-care behavior. To do so, modern medicine uses a supervised machine learning approach (ML) to study the psychosocial domains of frailty in cardiac patients with heart failure (HF). This study aimed to determine the absolute and relative diagnostic importance of the individual components of the Tilburg Frailty Indicator (TFI) questionnaire in patients with HF. An exploratory analysis was performed using machine learning algorithms and the permutation method to determine the absolute importance of frailty components in HF. Based on the TFI data, which contain physical and psychosocial components, machine learning models were built based on three algorithms: a decision tree, a random decision forest, and the AdaBoost Models classifier. The absolute weights were used to make pairwise comparisons between the variables and obtain relative diagnostic importance. The analysis of HF patients’ responses showed that the psychological variable TFI20 diagnosing low mood was more diagnostically important than the variables from the physical domain: lack of strength in the hands and physical fatigue. The psychological variable TFI21 linked with agitation and irritability was diagnostically more important than all three physical variables considered: walking difficulties, lack of hand strength, and physical fatigue. In the case of the two remaining variables from the psychological domain (TFI19, TFI22), and for all variables from the social domain, the results do not allow for the rejection of the null hypothesis. From a long-term perspective, the ML based frailty approach can support healthcare professionals, including psychologists and social workers, in drawing their attention to the non-physical origins of HF.

## Introduction

There is ample scientific evidence that psychosocial deficits coexist with abnormal aging (sometimes referred to as frailty syndrome [FS]), and even indications of their direct impact on this phenomenon^1–3^. Older people diagnosed with frailty are at risk of prolonged and repeated hospitalizations, which are often the result of neglect in the psychosocial sphere. Limited support of relatives, loneliness, and memory deficits affect the timeliness of visits to the doctor, signaling disturbing changes and compliance with medical recommendations (e.g., taking medications). Additionally, numerous scientific studies indicate that in patients diagnosed with FS, there is a coexistence of symptoms of depression that progress with the development of the syndrome^4^. The above-mentioned factors are particularly important in cardiac patients with heart failure (HF), who constitute a large group of patients diagnosed with FS^5^.

Using the principle of utilitarianism in scientific research, the main aim of this work was to study the absolute and relative diagnostic importance of variables from the Tilburg Frailty Indicator (TFI) frailty questionnaire^6,7^ for FS in a population of patients with HF. From a long-term perspective, insights from this study can inform psychologists, doctors, and social workers in preventive, diagnostic, and planning activities in the field of holistic treatment of the elderly and the organization of social assistance. Thus, the project will allow us to obtain psychosociological knowledge aimed at supporting medical personnel in dealing with patients and prognosing the effects of psychological and sociological deficits.

A review of contemporary research shows that psychological and social factors play an important role in the occurrence of FS and HF. Regardless of one’s view on the scientific problem—considering the psychosocial effects of cardiovascular disease and the patient’s weakness or perceiving them as one of the main causes—scientific evidence confirms that these phenomena coexist^8,9^. The importance of psychosocial factors in FS has been widely discussed in the literature. A relationship between depression and FS has been noted in numerous studies^4^. Studies of elderly women suffering from depression and displaying frailty symptoms, such as fatigue and slow walking, have shown increased mortality in this group^10^. However, there are reports that the relationship between frailty and depression is associated with depression only in the female sex, which may result in underestimation of symptoms among men due to cultural reasons and the inadequacy of depression research tools that differentially capture the patterns for each sex^11,12^.

In the case of sociological data related to the social status of individuals, variables related to education, material, and housing situations, as well as family situations, were mainly studied in the literature. Research on the female population in the United States shows a relationship between socioeconomic status and FS. A low level of education and lower income were significant, irrespective of the ethnicity of the surveyed women. People with lower education and income were more often diagnosed as “frail”^13^.

Cardiac patients constitute a very large group among the population of patients diagnosed with frailty. Researchers have used FS tools for certain types of HF^14^. People with HF are at greater risk of frailty, which may impair their self-care capabilities and lead to worsening symptoms of HF^15^. The reduced ability to take care of oneself, which may indirectly result from the psychosocial resources of patients, may be an additional factor in rehospitalization and increased mortality. Based on previous studies, researchers speculate that elderly HF patients also have difficulties taking care of themselves due to cognitive deficits. Both low self-care and impaired cognitive functioning are correlated with increased age, living alone, lower education levels, prolonged duration of illness, and a greater number of hospitalizations^16^. Further, both frailty and cognitive impairment often coexist with HF^5^. Studies have shown that the combined outflow of depression and physical frailty may increase the risk of cognitive impairment in patients with HF^17^. In cardiac patients with HF and FS, increased levels of anxiety and depression have been noted with increasing frailty^18^.

Despite the widely proven relationship between psychosocial factors and FS, it is impossible to ignore studies that have not shown such a relationship. For example, in a study carried out in the Netherlands, no evidence was found for the influence of psychosocial factors on the effects of FS: mortality and decline in performance in elderly patients^19^. However, the researchers stressed that longer studies may be needed to find differences and assumed that psychosocial factors in the prevention of FS may be of greater importance in the initial stages^19^. However, the Netherlands study attempted to obtain evidence of dependence, and in many of the studies presented below, we instead discuss the coexistence of the phenomena. This coexistence has been proven for frailty and for many psychological variables, including personal development, positive relationships, dealing with the environment, and self-acceptance. However, such coexistence was not found for variables such as a sense of independence in relation to one’s opinions or life goals^20^, indicating the ambiguity of the influence and coexistence of psychosocial factors on frailty.

Patients with FS and a comorbid condition, such as HF, are more likely to be affected by psychosocial deficits. Frequent hospitalizations resulting from difficulties in complying with medical recommendations may be the result of worsening memory processes, low mood, and loneliness, as well as a lack of care on the part of the patient’s relatives. All these psychosocial factors directly worsen the health of cardiac patients.

The aim of this study was to compare the importance of all psychosocial variables from the TFI questionnaire with selected variables (TFI responses) of the physical domain, that is, walking difficulties, lack of hand strength, and physical fatigue in patients with diagnosed HF. The choice of these three physical domain variables was driven by the characteristics of FS present in the elderly population. In the modern literature, FS includes characteristic features, such as a slowing down of gait^21^, a weakening of grip strength^22^, and a general feeling of physical fatigue^23^. The results of the absolute validity of all TFI variables are also presented.

## Methods

### Study design

The data were collected in line with ethical and medical standards by the medical staff of the University Teaching Hospital in Wroclaw from 2016 to 2019. The file did not contain any personal data that enabled the identification of patients. The study was approved by the Bioethics Committee of Wroclaw Medical University, Poland (KB–22/2018) and was performed in accordance with the Declaration of Helsinki. Informed consent was obtained from all study participants and/or their legal guardians. The participants (N = 666) included in the database were diagnosed with HF—562 were diagnosed with FS (coding: 1), and the remaining 104 were not diagnosed with FS (coding: 0).

### Research instruments

The FS of the patients was assessed using the Tilburg Frailty Indicator (TFI) questionnaire. The TFI questionnaire consists of two parts: frailty determinants and frailty components. The first part contains sociodemographic data, while the second part covers physical, psychological, and social components^7^. The eight components of the TFI that belong to the physical domain are physical unhealthiness, unintentional weight loss, walking difficulties, difficulty in maintaining balance, poor hearing, poor vision, lack of strength in the hands, and physical tiredness (TFI11–TFI18). The psychological domain includes four components: problems with memory, a drop in mood, agitation and irritability, and inability to cope with problems (TFI19–TFI22). The social domain of the TFI contains three components: living alone, lack of social relations, and lack of social support (TFI23–TFI25) (see Table 1).

**Table 1:** The specific items of the TFI questionnaire addressing subjective perceptions of physical difficulties and psychosocial problems^7^.

Item designation	TFI item
Physical components	
TFI11	Do you feel physically healthy?
TFI12	Have you lost a lot of weight recently without wishing to do so?
TFI13	Do you have difficulty in walking?
TFI14	Do you have difficulty maintaining your balance?
TFI15	Do you have poor hearing?
TFI16	Do you have poor vision?
TFI17	Do you have lack of strength in your hands?
TFI18	Do you have physical tiredness?
Psychological components	
TFI19	Do you have problems with your memory?
TFI20	Have you felt down during the last month?
TFI21	Have you felt nervous or anxious during the last month?
TFI22	Are you able to cope with problems well?
Social components	
TFI23	Do you live alone?
TFI24	Do you sometimes miss having people around you?
TFI25	Do you receive enough support from other people?

### Exploratory analysis

**Figure 1:**
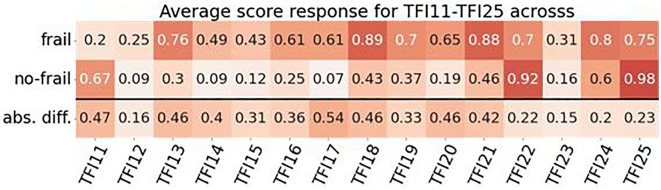
The average values of the positive responses and the value of the absolute difference in FTI11-TFI25 in frail and non-frail patients.

The work analyzed questions from the TFI questionnaire (15 questions concerning physical and psychosocial components: TFI11–TFI25). Figure 1 below shows the average values of the positive responses and the value of the absolute difference between them. The absolute difference is necessary because of inverted questions whose values did not change during data preparation (TFI11, TFI22, and TFI25). Positive responses for physical (TFI11–TFI18), psychological (TFI19–TFI22), and social (TFI23–TFI25) were found to have disproportionate scores for both groups. Some of the positive answers had a high average in the group of people without frailty syndrome, and this value increased relatively slightly in the group of frail patients (e.g., TFI23 0.31–0.16 = 0.15; for comparison, TFI17 0.61–0.07 = 0.54). These results became a premise for further research related to the analysis of the diagnostic importance of individual TFI variables.

The results of the exploratory analysis, supported by pairwise comparison methods, were used to obtain answers to the research questions concerning the individual diagnostic criteria (variable importance) of TFI. It was limited to comparing psychosocial variables with three selected variables from the physical domain, which resulted in 21 comparisons.

To determine the absolute importance of the explanatory variables in the machine learning classification models, the permutation method was used^24^. The absolute weightings were then used to determine the relative diagnostic importance. To assess the statistical significance between the differences in the importance of the variables, post hoc multiple comparisons were made using a t-test. Statistical significance for relative values was determined using Bonferroni correction^25^.

The value of the *z* statistic allowed us to determine the significance of the selected variable (however, since the *z* statistic depends on the number of permutations, it is easier to follow the *p* value). In the case of the importance of absolute variables, a one-tailed test was used, whereas for comparisons between pairs of variables, a two-tailed test was performed to assess the absolute value of the *z* statistic. The interpretation of the sign indicated which of the compared variables had a greater diagnostic value. These statistics were translated into the *p* value.

### Machine learning

For the purposes of machine learning, the sample was divided into subsets: training, validation, and testing. After missing data were discarded, the sample of 666 patients was limited to 612 patients (4/6 of these were used for the training procedure, 1/6 for validation, and the remaining 1/6 for the testing activities). Data division was performed using a randomizing function.

Based on the above theoretical assumptions and the purpose of the research, the following machine learning models were used, as implemented in the Scikit-learn statistical package (https://scikit-learn.org): decision tree, random forest, and AdaBoost classifier. During the training phase, the models were trained on the training set (4/6 of the data) with different hyperparameters, and their performance was evaluated on the validation data (1/6 of the data). Hyperparameters of the models were selected based on the validation scores during this phase in such a way that they did not favor either true positive or true negative values (in other words, true positive and true negative values were treated on an equal footing). In the case of many possible configurations, the model with the lowest complexity was selected from the heatmap. Following these rules, a model with a data fraction per leaf parameter of 0.1 (minimum sample per leaf, calculated as a data fraction) and a depth of 3 (maximum depth) was selected for the decision tree algorithm. On the validation subset, this model achieved a detection accuracy level of 88%, both for people with a diagnosis of frailty and for those without a frailty diagnosis. Models with these parameters were then subjected to a test procedure.

For a random forest, a model with a data fraction per leaf parameter of 0.03 (minimum sample per leaf) and a depth of 4 (maximum depth) was selected. This model achieved, on the validation subset, the correct detection of people with FS at the level of 95% and people without an FS diagnosis at a similar level of 94%. The number of trees was arbitrarily set to 100.

For the AdaBoost classifier, a model with parameters of 200 (number of trees) and 1 (maximum depth) was selected. On the validation subset, this model achieved a detection accuracy level of 100%, both for people with FS and for those without FS.

### Ethical approval

The study was approved by the Bioethics Committee of Wroclaw Medical University, Poland (KB–22/2018).

### Consent to participate

Informed consent was obtained from all study participants and/or their legal guardians.

## Results

Table 2 shows the characteristics of the sample. There was a large disproportion between the number of patients diagnosed with FS (N = 562) and those without an FS diagnosis (N = 104). The mean age was higher by approximately five years in patients with FS (73 years) compared to patients without FS (68 years). The comparison of the percentage of single people with a diagnosis of FS and without FS (47.2% and 63.5%, respectively) showed a clear disproportion between the two groups. In the group of people without FS, more patients exceeded the income equal to PLN 2101 (28.8%) than in the group of people diagnosed as frail (21.0%).

**Table 2:** Characteristics of the sample.

Character	Total N = 666 (100%)	Frail N = 562 (84.4%)	Non-frail N = 104 (15.6%)
Gender			
Male	359 (53.9%)	295 (52.5%)	64 (61.5%)
Female	307 (46.1%)	267 (47.5%)	40 (38.5%)
Age			
Mean; SD	72.07; 10.97	72,88; 10.97	67,68; 10.79
Relationship			
Lonely	335 (50.3%)	297 (52.8%)	38 (36.5%)
In relationship	331 (49.7%)	265 (47.2%)	66 63.5%)
Education level			
Primary school	207 (31.1%)	190 (33.8%)	17 (16.3%)
Basic vocational	60 (9.0%)	54 (9.6%)	6 (5.8%)
High school	295 (44.3%)	242 (43.0%)	53 (51.0%)
University degree	103 (15.5%)	75 (13.5%)	28 (26.9%)
Monthly income Min. mode	148 (22.2%)	118 (21.0%)	(28.8%)
(PLN)	2101	2101	2101
Other diseases			
Diabetes	280 (42.1%)	251 (44.6%)	29 (4.3%)
Hypertension	513 (77.1%)	442 (66.3%)	71 (10.6%)
Both	235 (35.3%)	211 (31.7%)	24 (3.6%)

*SD* standard deviation, *N* number of participants, *PLN* Polish zloty (EUR 1 = PLN 4.64 in 2021).

Table 3 shows the true positive and true negative test values for the machine learning models built and verified in the previous phases (training and validation). The decision tree yielded 79% true positive and 87.5% true negative values; that is, the classification ability of this model was higher for people without FS than for people diagnosed with frailty. Learning with a random forest of decision trees led to a deterioration in the recognition of people with FS (93%) compared to those without FS (100%), but in the case of this model, the overall classification accuracy was much higher than that obtained by the decision tree. The AdaBoost algorithm was the only classifier that allowed better recognition of a person diagnosed with frailty (100%) than people without FS (93.8%), and it showed an overall classification accuracy similar to that of a random decision forest.

**Table 3:** True positives and true negatives values for the three ML algorithms obtained in testing phase.

	TPR (%)	TNR (%)
Decision tree	79.07	87.50
Random forest	93.02	100.00
AdaBoost	100.00	93.75

*TPR* true positive rate, *TNR* true negative rate.

### Absolute diagnostic importance of TFI variables for the sample of patients with HF

**Figure 2:**
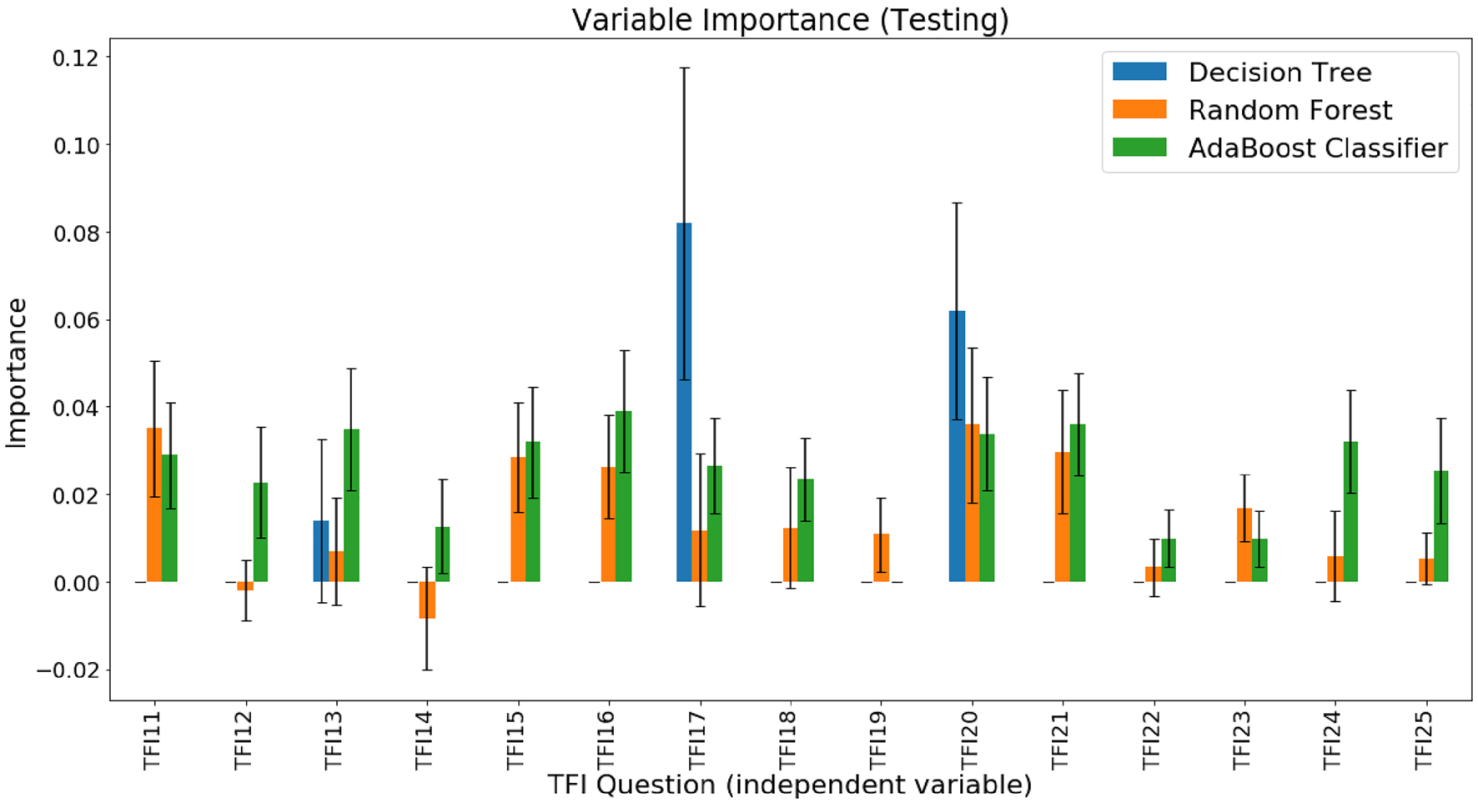
Absolute diagnostic importance of the variables based on the test subset—mean value (colored bars) and standard deviation (gray line).

**Figure 3:**
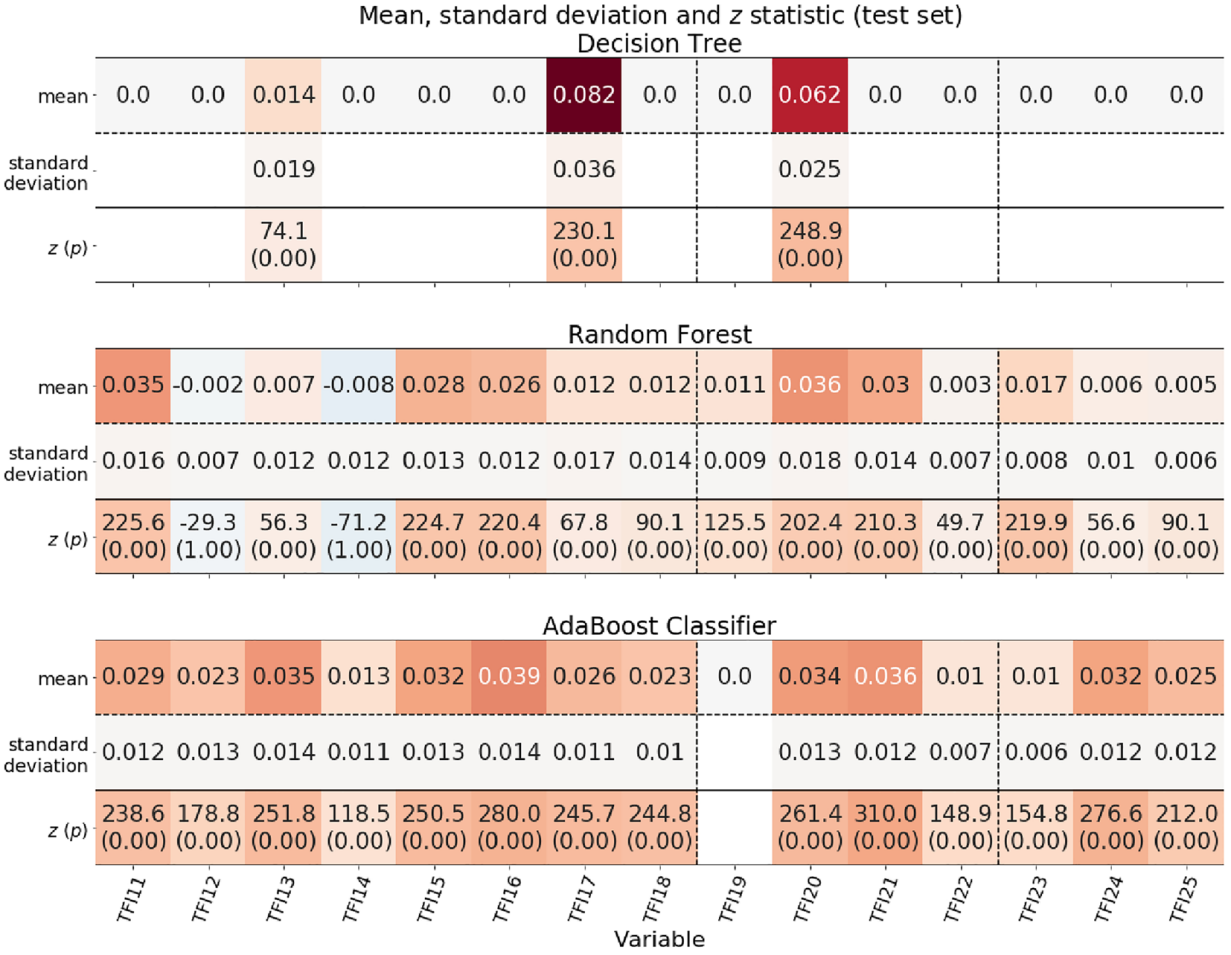
Absolute diagnostic validity of TFI variables and the corresponding statistical significance test.

In Fig. 2 and in the corresponding Fig. 3, which variables in particular models turned out to be important was assessed. It should be remembered that achieving *p* values higher than 0.05 does not necessarily imply that the model did not use the given variable. The diagnostic importance of the variables was measured by changes in the accuracy of the model, as described in the subsection on the assessment of the importance of the explanatory variables.

At the testing stage, the use of the decision tree resulted in the physical variable TFI17 achieving diagnostic significance (*µ* = 0.082, *σ* = 0.036, *z* = 230.1, *p* = 0.00), and the psychological variable TFI20 achieved diagnostic significance (*µ* = 0.062, *σ* = 0.025, *z* = 248.9, *p* = 0.00), while for the physical variable TFI13, the significance was *µ* = 0.014 (*σ* = 0.019, *z* = 74.1, *p* = 0.00). With the random decision forest, the following variables were of greatest diagnostic importance: TFI20 (*µ* = 0.036, *σ* = 0.018, *z* = 202.4, *p* = 0.00), TFI11 (*Do you feel healthy physically?*; *µ* = 0.035, *σ* = 0.016, *z* = 225.6, *p* = 0.00), and TFI21 (*µ* = 0.030, *σ* = 0.014, *z* = 210.3, *p* = 0.00). For the AdaBoost classifier, the most significant component was that of the physical domain—TFI16 (*µ* = 0.039, *σ* = 0.014, *z* = 280.0, *p* = 0.00)—followed by the variables in the psychological domain—TFI21 (*µ* = 0.036, *σ* = 0.012, *z* = 310.0, *p* = 0.00), TFI13 (*µ* = 0.035, *σ* = 0.014, *z* = 251.8, *p* = 0.00), and TFI20 (*µ* = 0.034, *σ* = 0.013, z = 261.4, *p* = 0.00)—the sociological variable TFI24 (*µ* = 0.032, *σ* = 0.012, *z* = 276.6, *p* = 0.00), and the variable TFI15 belonging to the physical domain (*Do you experience difficulties due to poor hearing on a daily basis?*; *µ* = 0.032, *σ* = 0.013, z = 250.5, *p* = 0.00). The explanatory variable TFI19 turned out to be irrelevant for the model on this subset of data (*µ* = 0).

### The relative diagnostic importance of TFI variables for a sample of patients with HF

**Figure 4:**
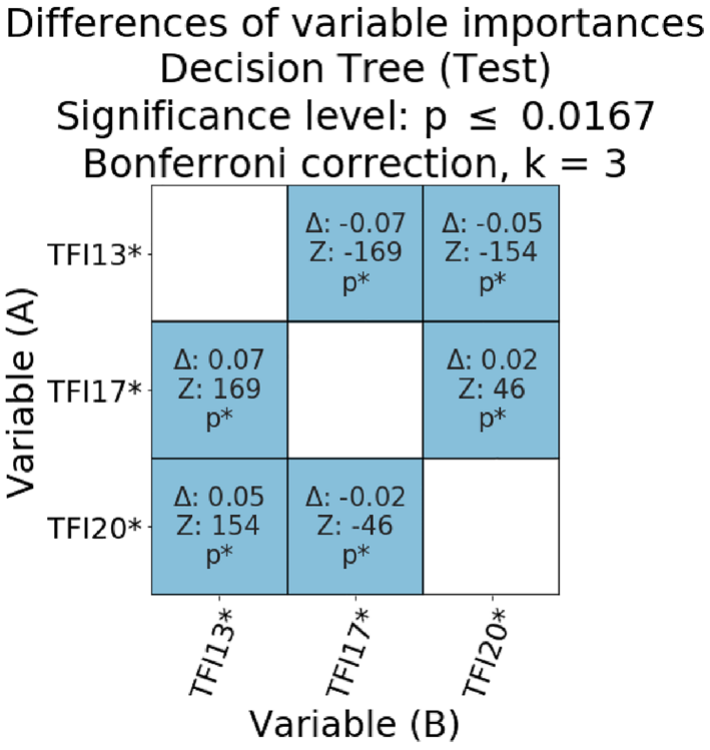
Matrix of relative importance of diagnostic variables for the test subset in the decision tree.

**Figure 5:**
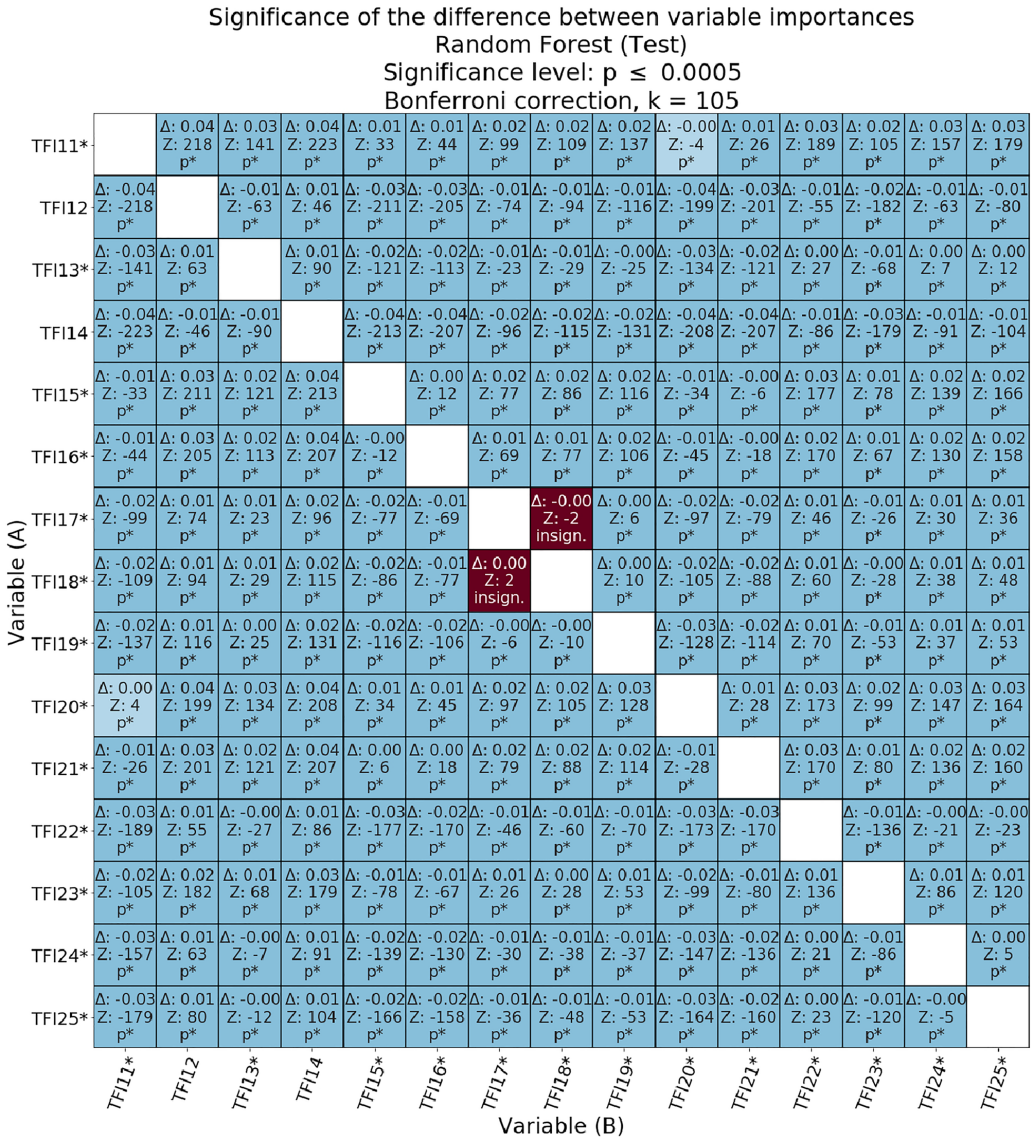
Matrix of relative importance of diagnostic variables for the test subset in a random decision forest.

**Figure 6:**
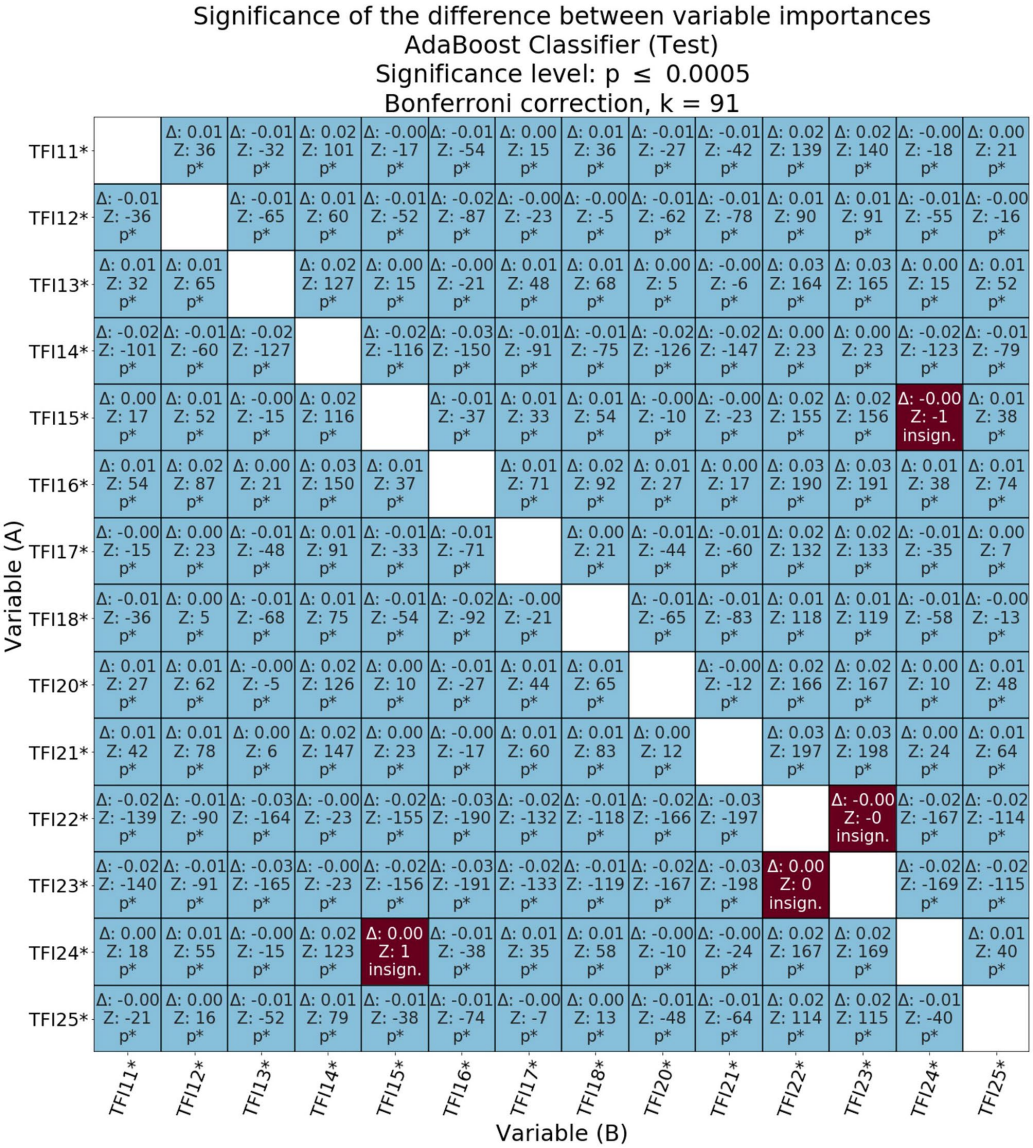
Matrix of relative importance of diagnostic variables on the basis of the test subset in the AdaBoost classifier.

Figures 4, 5, 6 present the relative statistical significance of individual variables according to individual models. Since we were dealing with multiple comparisons, the *p*-values were calculated using Bonferroni correction. The statistically significant corrected values are marked in the tables with the symbol p*. The differences (Δ) between the importance of the variables and the statistical significance of these differences were also determined. Statistical significance for the comparisons of the meanings of individual pairs of variables was calculated as described in the methodological section. If the value of Δ was negative, it meant that in the validity comparison, the A-axis variable was diagnostically “less important” than the B-axis value. Moreover, if the Δ value was positive, it meant that the A-axis variable was diagnostically more important for model classification from the variable on the B-axis. Marking in the form of an asterisk on the A and B axes of significance (for *p* < 0.05) refers to absolute values.

Considering the classification accuracy of individual models, the AdaBoost and random forest algorithms had similar accuracies; thus, both models were selected *ex aequo* to verify the hypotheses, whereas the decision tree model was rejected due to lower accuracy and is presented for illustrative purposes only.

Figure 4 shows the relative diagnostic importance of variables in the decision tree model based on the test subset. All TFI variables used to build this model were important in the analysis of absolute values; therefore, all possible pairs of variables were compared. TFI17 was more important than TFI20 (Δ = 0.02, *z* = 46, *p* ≤ 0.0167) and TFI13 (Δ = 0.07, *z* = 169, *p* ≤ 0.0167). The relative difference between TFI20 and TFI13 was Δ = 0.05 (*z* = 154, *p* ≤ 0.0167). Based on the results obtained with the decision tree model, the psychological variable TFI20 (“*Have you experienced a drop in mood over the last month?*”) was indicated as the second most important factor for the correct classification of people with FS. However, as many as two of the three variables used in the model belonged to the physical components of the TFI questionnaire.

The relative diagnostic importance of the variables for the test subset with a random forest is presented in Fig. 5. The differences between the importance of the variables for the AdaBoost algorithm on the test subset are presented in Fig. 6. Based on the hypotheses put forth in the study regarding the comparison of physical components with psychosocial components, comparisons between selected physical components (TFI13, TFI17, and TFI18) and psychosocial components (TFI19–TFI25) were critical.

The diagnostic validity of the psychological variable TFI19 (*memory problems*) obtained using a random forest was significantly higher than that of TFI13 (*walking difficulties*; Δ = 0.004, *z* = 25, *p* ≤ 0.0005), lower than TFI17 (*lack of hand strength*; Δ = 0.001, *z* = − 6, *p* ≤ 0.0005), and lower than TFI18 (*physical fatigue*; Δ = 0.002, *z* = − 10, *p* ≤ 0.0005). In the case of the AdaBoost classifier, TFI19 did not participate in the construction of the model, indicating that for a specific subset of data, this variable did not contribute; that is, it did not affect the classification accuracy of FS. The consequence of this is that each of the other variables was diagnostically more important than TFI19. As a result of the above analysis, there is insufficient evidence to conclude that the psychological variable TFI19 is diagnostically more important than TFI13 (*walking difficulties*) or less important than TFI17 (*lack of hand strength*) and TFI18 (*physical fatigue*).

The diagnostic validity of the next psychological variable TFI20 (*Have you experienced a drop in mood during the last month?*) obtained with the use of a random forest was significantly greater than TFI13 (*walking difficulties*, Δ = 0.029, *z* = 134, *p* ≤ 0.0005), TFI17 variable (*lack of hand strength*, Δ = 0.024, *z* = 97, *p* ≤ 0.0005) and TFI18 (*physical fatigue*, Δ = 0.023, *z* = 105, *p* ≤ 0.0005). The AdaBoost classifier showed that the diagnostic importance of TFI20 was statistically significantly lower than that of TFI13 (*walking difficulties*, Δ = 0.001, *z* = − 5, *p* ≤ 0.0005), but higher than TFI17 (*lower hand strength*, Δ = 0.007, *z* = 44, *p* ≤ 0.0005) and TFI18 (*physical fatigue*, Δ = 0.010, *z* = 65, *p* ≤ 0.0005). Thus, there is insufficient evidence to conclude that the psychological variable TFI20 is diagnostically more important than TFI13 (*walking difficulties*). However, both algorithms unequivocally confirmed that the variable TFI20 is diagnostically more important than TFI17 and TFI18: lack of hand strength and physical fatigue, respectively.

For the psychological variable TFI21 (*Have you felt nervous or excited during the last month?*), the diagnostic importance obtained using a random forest was significantly greater than TFI13 (*walking difficulties*, Δ = 0.023, *z* = 121, *p* ≤ 0.0005), TFI17 (*lack of hand strength*, Δ = 0.018, *z* = 79, *p* ≤ 0.0005), and TFI18 (*physical fatigue*, Δ = 0.017, *z* = 88, *p* ≤ 0.0005). The AdaBoost classifier showed that the diagnostic importance of TFI21 was statistically significantly higher than that of TFI13 (*walking difficulties*, Δ = 0.001, *z* = 6, *p* ≤ 0.0005), TFI17 (*lower hand strength*, Δ = 0.009, *z* = 60, *p* ≤ 0.0005) and TFI18 (*physical fatigue*, Δ = 0.013, *z* = 83, *p* ≤ 0.0005). Ultimately, the psychological variable TFI21 was revealed to be diagnostically more important than the selected physical variables: walking difficulties, lower hand strength, and physical fatigue.

The diagnostic importance of the fourth psychological variable, TFI22 (*Can you deal with problems?*), obtained using a random forest, turned out to be significantly lower than TFI13 (*walking difficulties*, Δ = 0.004, *z* = − 27, *p* ≤ 0.0005), TFI17 (*lower hand strength*, Δ = 0.009, *z* = − 46, *p* ≤ 0.0005) and TFI18 (*physical fatigue*, Δ = 0.009, *z* = − 60, *p* ≤ 0.0005). The AdaBoost classifier showed that the diagnostic validity of TFI22 was significantly lower than that of TFI13 (*walking difficulties*, Δ = 0.025, *z* = − 164, *p* ≤ 0.0005), TFI17 (*lower hand strength*, Δ = 0.017, *z* = − 132, *p* ≤ 0.0005) and TFI18 (*physical fatigue*, Δ = 0.014, *z* = − 118, *p* ≤ 0.0005). Thus, the psychological variable TFI22 was diagnostically less important than the three variables from the physical domain: walking difficulties, lack of hand strength, and physical fatigue.

The importance of the sociological variable TFI23 (*Do you live alone?*) with a random decision forest turned out to be diagnostically greater than that of TFI13 (*walking difficulties*, Δ = 0.010, *z* = 68, *p* ≤ 0.0005), TFI17 (*lack of hand strength*, Δ = 0.005, *z* = 26, *p* ≤ 0.0005) and TFI18 (*physical fatigue*, Δ = 0.004, *z* = 28, *p* ≤ 0.0005). The AdaBoost classifier showed that the diagnostic importance of the sociological variable TFI23 was much lower than that of TFI13 (*walking difficulties*, Δ = 0.025, *z* = − 165, *p* ≤ 0.0005), TFI17 (*lack of hand strength*, Δ = 0.017, *z* = − 133, *p* ≤ 0.0005,) and TFI18 (*physical fatigue*, Δ = 0.014, *z* = − 119, *p* ≤ 0.0005). In the case of the AdaBoost classifier, there was a similarity between the comparative results of TFI22 and TFI23 against all other variables. However, for TFI23, based on the random forest and AdaBoost results, it is impossible to unequivocally determine the relative diagnostic importance of this variable over the three physical variables: walking difficulties, lack of hand strength, and physical fatigue.

For the sociological variable TFI24 (*Do you ever miss the company of other people?*), its diagnostic importance in a random forest was significantly lower than that of TFI13 (*walking difficulties*, Δ = 0.001, *z* = − 7, *p* ≤ 0.0005), TFI17 (*lower hand strength*, Δ = 0.006, *z* = − 30, *p* ≤ 0.0005) and TFI18 (*physical fatigue*, Δ = 0.007, *z* = − 38, *p* ≤ 0.0005). The AdaBoost classifier showed that the diagnostic importance of TFI24 was statistically significantly lower than that of TFI13 (*walking difficulties*, Δ = 0.003, *z* = − 15, *p* ≤ 0.0005), but greater than that of TFI17 (*lower hand strength*, Δ = 0.006, *z* = 35, *p* ≤ 0.0005) and TFI18 (*physical fatigue*, Δ = 0.009, *z* = 58, *p* ≤ 0.0005). Thus, it is concluded that the sociological variable TFI24 is less important than the variable walking difficulties. There was no clear evidence to confirm the hypothesis that TFI24 is more important than the physical variables: lack of strength in the hands and physical fatigue.

The importance of the third (and last) sociological variable, TFI25 (*Do you receive a lot of support from others?*), in a random decision forest was significantly less diagnostically important than TFI13 (*walking difficulties*, Δ = 0.002, *z* = − 12, *p* ≤ 0.0005), TFI17 (*lower hand strength*, Δ = 0.007, *z* = − 36, *p* ≤ 0.0005) and TFI18 (*physical fatigue*, Δ = 0.007, *z* = − 48, *p* ≤ 0.0005). The AdaBoost classifier showed that the diagnostic importance of TFI24 was less diagnostically important than that of TFI13 (*walking difficulties*, Δ = 0.009, *z* = − 52, *p* ≤ 0.0005), TFI17 (*lower hand strength*, Δ = 0.001, *z* = − 7, *p* ≤ 0.0005) and TFI18 (*physical fatigue*, Δ = 0.002, *z* = 13, *p* ≤ 0.0005). The sociological variable TFI25 was found to be less important than the physical variable linked to walking difficulties. There was no clear evidence to confirm the hypothesis that the variable TFI25 is more important than the other two variables from the physical domain: lack of strength in the hands and physical fatigue.

It is noteworthy that the difference between determining the importance of variables by the AdaBoost and random forest models, which, despite similar accuracy results, did not assign similar importance to all variables (see Fig. 3). This may be because the random decision forest achieved better results for truly negative values (i.e., in detecting persons without FS) and the AdaBoost classifier for true positive values (detecting frail persons), that is, differences in the algorithms themselves.

## Discussion

The results of the machine learning models showed that some of the psychological questions from the TFI questionnaire had very high absolute diagnostic validity. In the case of relative diagnostic importance, when checking whether the variables from the psychological and social components were more important than the physical components, the most interesting for us were pairs of variables consisting of one question belonging to the physical component and one question belonging to the psychosocial components. Questions that did not differ significantly from each other indicated non-significant differences between these variables, and the variables that differed by very small values indicated very similar importance.

To verify the hypotheses, the results of the two best algorithms were considered—the random forest and the AdaBoost classifier. A pairwise comparison of components from the physical domain with psychosocial components was made for three selected components characteristic of FS: TFI13, TFI17, and TFI18. The psychological variable TFI20 was found to be more diagnostically important than the other variables from the physical domain: TFI17 and TFI18. Further, the psychological variable TFI21, which diagnosed the patient’s irritability, turned out to be diagnostically more important than all three physical variables considered: TFI13, TFI17, and TFI18. In the case of the other two variables from the psychological domain (TFI19 and TFI22) as well as all variables from the social domain, the obtained results do not allow for the rejection of the null hypothesis. This could be because these variables were less diagnostically important in relation to the three variables analyzed from the physical domain, or because the two best algorithms assigned different diagnostic importance to these variables.

The analysis of the results concerning psychological variables showed that a feeling of irritability (nervousness or excitement) over the last month was the most important diagnostic question among all psychosocial TFI questions. This highlights the problem of stress in people with FS among the population of patients with HF. By contrast, a drop in mood over the last month, which was more diagnostically important than TFI17 and TFI18, may indicate a risk of depression in people with FS in the cardiology group studied. The results show the functioning deficits of the population of people with FS and are consistent with the results of the current research.

It is interesting that variable TFI19, diagnosing memory problems characteristic of old age, was not recognized by any of the models as one of the most important variables, and was not used at all two of them—the decision tree and the AdaBoost classifier. Moreover, TFI22, the ability to deal with problems, although used to construct the two best machine learning models, was not more diagnostically important than any of the three physical components analyzed.

Social variables did not gain a clear diagnostic advantage over variables from the physical domain. However, TFI24, diagnosing the need for relationships with other people, was one of the most important diagnostically important variables in the AdaBoost classifier. The ambiguity of the results obtained for this variable indicates the need for a deeper analysis of the phenomenon of longing related to relationships with other people. The reverse situation occurred in the case of TFI23 because it was considered one of the most important diagnostically in the random decision forest algorithm but not by the AdaBoost algorithm. In addition, in this case, research on the importance of patients living alone should be intensified. The phenomenon of loneliness is currently a subject of interest in research on FS^26^.

Notably, the differences between the validation and test results between the same models may indicate differences in subsets that could not be avoided, even with the randomization process. Although all data should be analyzed, test models are the most important for model evaluation and drawing conclusions. The differences between the validation and the test usually indicate the existence of the model overfitting effect; thus, future research should minimize these differences using measures that overcome this problem. One such strategy would be to use more data and repeat the training, validation, and testing processes. Another option is to use an already existing model from the validation and then run only the test process on the new data, which is less time consuming but does not guarantee an improved model (if it is obtainable). The process of dividing the variables into training, validation, and test subsets could also be repeated to ensure that these subsets differ as little as possible.

The main aim of this work was to develop a pilot study using existing frailty testing tools to find the most diagnostically important variables of this syndrome in the population of patients with HF. In the long term, this knowledge could be used by doctors, psychologists, and social workers, drawing attention to the biggest problems in the non-physical sphere of patients. Another motivation for the study was the possibility of developing an improved frailty testing tool adapted to the symptoms of patients with HF. We should not exclude the possibility that for a specific group of patients—for example, cardiac patients with FS—there are some relationships between variables that are less noticeable for the general population of patients diagnosed with frailty. Analyses of this type were not, however, the aim of this study, as for this purpose, it would be necessary to consider additional patient populations without HF.

Identifying the psychosocial factors related to the adverse condition of frail patients with HF is crucial for improving care quality and reducing costs. Existing psychosocial factor identification approaches are primarily based on guidelines or observational data on frailty. Our ML-based frailty algorithm as a possible module of the healthcare analytics system can identify psychosocial variables that are not in the existing known factors. This intelligent solution enables patient care analytics, where valuable and relevant information about patients is required in real-time by asking medical staff questions to the patients they care for. For instance, our study showed that experiencing feelings of irritability (nervousness or excitement) over the past month proved to be the most diagnostically important question of all the TFI psychosocial items. This result draws attention to the problem of stress in people with heart failure. On the other hand, the decrease in mood over the last month, which is diagnostically more important than physical components of FS (the TFI17 and TFI18), may indicate the risk of depression in this cardiac patient population. The proposed machine learning approach can infer such implicit information on the psychological condition of HF patients and extract relevant information for recommending psychological interventions.

Another important premise of this work was the analysis of the risk of an incorrect diagnosis based on incomplete information about the patient. In the era of research focused on large datasets, despite efficient diagnostic systems, medical and social services must make immediate decisions based on limited, incomplete patient data. This is especially true of semi-open diagnostic systems, where a diagnosis can be given with the likelihood of the diagnosis being correct, depending on the data, the quantity of which may be limited, or when creating traditional, abbreviated versions of the questionnaires. Thus, specialists could receive valuable hints regarding what information or variables can be replaced with other information or variables in the model.

This work is a contribution to future research, allowing us to verify the impact of individual variables on the appearance of FS and its health over many years, which may become possible with increasingly developed panel databases. It should also be emphasized that although this work did not attempt to build a model for patients with pre-frail syndromes, the approach presented in this paper shows how subtle differences in multivariate models can be captured. Such an approach will be used in the near future for preventive purposes in the process of holistic treatment. Psychologists could be involved in this process, inter alia, thereby gaining valuable knowledge about the patient population and the areas of necessary influence.

## Conclusion

This study analyzed the diagnostic validity of the psychosocial and physical criteria used in the diagnostic process of frail patients in an elderly population suffering from HF. Following the AI approach and the TFI survey data, including the physical, psychological, and social domains, three machine learning models were constructed based on separate algorithms of the decision tree, random decision forest, and AdaBoost classifier. The findings show that none of the variables belonging to the social domain were more diagnostically important than the variables from the physical domains linked to experiencing difficulties due to walking difficulties, lack of hand strength, and physical fatigue. In the case of psychological criteria for FS, the factors of irritability were diagnostically more important than all three analyzed physical variables, while the variable of depression mood was diagnostically more important than the variables related to physical weakness, that is, lack of hand strength and physical fatigue.

## Limitation

The study has some limitations. Firstly, since the effects of psychological and sociological deficits (coexistence of phenomena) are shown, the ambiguity of the influence and coexistence of psychosocial factors on frailty is possible. Therefore, there may be unclear conclusions about the impact of psychosocial factors and their dependencies on frailty. This, in turn, might implicate suggest unknown causal dependencies between disease and psychosocial deficits. Psychological and social deficits are part of the frailty model. As these deficits are included in the operational definition of frailty, the Tilburg Frailty Indicator (TFI) embraces such measures. According to the conceptual model of frailty^27^, multidimensional frailty along with physical, psychological, and social components should be considered a consequence of individual diseases and multimorbidity. Furthermore, the use of machine learning does not solve the problem of causal inference in observational datasets. Algorithms can be useful in predicting outcomes, but predictors per se do not explain causality. The second limitation were input hypotheses. The number of the hypotheses was limited to comparing psychosocial variables with three selected variables from the physical domain, which resulted in obtaining 21 comparisons. This selected input hypotheses were driven by clinical background of patients with heart failure. Obviously, further study may be unrestricted and embrace more hypothetical directions.

## Data Availability

The datasets generated during and/or analysed during the current study are available from the corresponding author on reasonable request.
